# Learning to predict expression efficacy of vectors in recombinant protein production

**DOI:** 10.1186/1471-2105-11-S1-S21

**Published:** 2010-01-18

**Authors:** Wen-Ching Chan, Po-Huang Liang, Yan-Ping Shih, Ueng-Cheng Yang, Wen-chang Lin, Chun-Nan Hsu

**Affiliations:** 1 Institute of Biomedical Informatics, National Yang-Ming University, Taipei, Taiwan; 2 Bioinformatics Program, Taiwan International Graduate Program, Academia Sinica, Taipei, Taiwan; 3 Institute of Information Science, Academia Sinica, Taipei, Taiwan; 4 Institute of Biological Chemistry, Academia Sinica, Taipei, Taiwan; 5 Institute of Biomedical Sciences, Academia Sinica, Taipei, Taiwan

## Abstract

**Background:**

Recombinant protein production is a useful biotechnology to produce a large quantity of highly soluble proteins. Currently, the most widely used production system is to fuse a target protein into different vectors in *Escherichia coli *(*E. coli*). However, the production efficacy of different vectors varies for different target proteins. Trial-and-error is still the common practice to find out the efficacy of a vector for a given target protein. Previous studies are limited in that they assumed that proteins would be over-expressed and focused only on the solubility of expressed proteins. In fact, many pairings of vectors and proteins result in no expression.

**Results:**

In this study, we applied machine learning to train prediction models to predict whether a pairing of vector-protein will express or not express in *E. coli*. For expressed cases, the models further predict whether the expressed proteins would be soluble. We collected a set of real cases from the clients of our recombinant protein production core facility, where six different vectors were designed and studied. This set of cases is used in both training and evaluation of our models. We evaluate three different models based on the support vector machines (SVM) and their ensembles. Unlike many previous works, these models consider the sequence of the target protein as well as the sequence of the whole fusion vector as the features. We show that a model that classifies a case into one of the three classes (no expression, inclusion body and soluble) outperforms a model that considers the nested structure of the three classes, while a model that can take advantage of the hierarchical structure of the three classes performs slight worse but comparably to the best model. Meanwhile, compared to previous works, we show that the prediction accuracy of our best method still performs the best. Lastly, we briefly present two methods to use the trained model in the design of the recombinant protein production systems to improve the chance of high soluble protein production.

**Conclusion:**

In this paper, we show that a machine learning approach to the prediction of the efficacy of a vector for a target protein in a recombinant protein production system is promising and may compliment traditional knowledge-driven study of the efficacy. We will release our program to share with other labs in the public domain when this paper is published.

## Background

Acquiring large quantities of a desired protein *in situ *from original host cells is not trivial. Moreover, gene over-expression and purification of corresponding proteins in a soluble form are important for structural and functional proteomics. Recombinant protein production is an important applicable procedure in biotechnology and one of the few ways to over-express a given protein coding sequence of interest. To date, *Escherichia coli *(*E. coli*), one of Gram-negative bacteria, is still an approachable and favored host for cloning and expressing a given protein in many occasions [1-3]. In recent years, a variety of studies have developed well-established large-scale and high-throughput systems to obtain large quantities of soluble recombinant proteins [4-6]. However, it has been reported that a large number of foreign heterologous proteins were expressed at relatively low levels [7] or difficult to solubilize [8], in *E. coli*. These over-expressed proteins in an insoluble form are termed as inclusion bodies. Since the refolding procedure of inclusion bodies to recover a soluble form of recombinant proteins is time-consuming, expression of insoluble protein aggregates is frequently a major obstacle in recombinant protein production.

Hence, to meet the demands of preventing inclusion body formation in recombinant expression systems, many researchers have dedicated their efforts to optimize the growth conditions, such as buffer composition, protein concentration, and cultivation temperature. While others have focused on improving the folding probabilities regarding enhancement of mRNA stability, over-expression of rare-codon tRNA, selection of efficient vectors and host strains, and co-expression with solubility-enhanced proteins [3]. Nevertheless, more studies have emphasized the importance of increasing the solubility of recombinant proteins in *E. coli *by fusing them to highly soluble carrier proteins [9-12]. Because the only way to select a specific match between a target protein and an appropriate fusion partner that will lead to a soluble form is still a trial-and-error process; a more systematic approach is required.

Regardless of fusing different vectors, most previous works have attempted to predict the propensity of a given protein to be soluble or not in *E. coli*. The first such study was conducted by Wilkinson and Harrison [13] with a regression model analysis. They concluded five amino acid-dependent factors are discriminative features that correlate to inclusion bodies formation. There were charge average approximation (Asp, Glu, Lys and Arg), turn-forming residue fraction (Asn, Gly, Pro and Ser), cysteine and proline fractions, hydrophilicity and molecular weight. In a subsequent study, Davis *et al*. have improved the statistical solubility model of solubility in *E. coli *by demonstrating that the first two parameters were more critical than other three [14]. Additionally, based on the undertaking of structural genomics projects, Bertone *et al*. have applied machine learning techniques such as decision trees and Support Vector Machines (SVMs) to discover other informative features based on 562 proteins from Methanobacterium thermoautotrophicum. Among these critical parameters, low content of negative residues (DE <17%) and presence of hydrophobic patches are associated with insoluble protein formation [15]. Subsequently, Goh *et al*. utilized random forest and decision-tree based methods on about 27, 000 protein targets in TargetDB [16] to conclude that the most significant protein feature was serine percentage composition [17]. Furthermore, Luan *et al*. collected 1, 536 soluble proteins out of 10,167 ORFs in *Caenorhabditis elegans *expressed by single vector and one *E. coli *strain. In their study, the most prominent protein feature was GRAVY (Grand Average of Hydropathicity, an indicator for average hydrophobicity of a protein) [18].

To date, many studies have showed that Support Vector Machines (SVMs) combining with appropriate kernels frequently result in better performance for biological sequence classification than other methods based on statistical learning theory [19,20]. Recently, many studies have tried to apply SVMs to circumvent the problem of assessing the propensity of target proteins to be actively soluble or to form inclusion body in *E. coli*. According to their previously observed sequence-dependent features in protein levels, Idicula-Thomas *et al*. provided a SVM-based approach to achieve 72% in prediction accuracy [21,22]. Additionally, Smialowski *et al*. developed PROSO, a two-layered predictor combining SVM and Naive Bayes classifiers, and obtained a compatible performance similar to Idicula-Thomas *et al*. [23].

The studies mentioned above have at least two basic postulations: 1) a given gene was thought to have been over-expressed and 2) the expression level of a target gene was the same whatever by fusing different vectors in *E. coli*. Consequently, most previous works only focused on demonstrating important factors related to solubility prediction and mixed the cases of target genes in inclusion fraction and non-expression to form a negative set. However, recent research has reported that recombinant proteins expressed as inclusion bodies still keep biological activity than previously appreciated [24]. Thus, it is still significant to distinguish inclusion bodies from the negative set in previous studies. Moreover, it has also been assumed that all given proteins obtained the same expression result regardless of the fusion of different vectors in *E. coli *as it only focused on predicting the solubility of a target gene by its protein sequence. However, a given protein usually yielded different expression levels by fusing different vectors. Note that different groups have discovered different crucial protein factors according to soluble target proteins acquired from their own experiments. It would be partly because their experimental data were conducted under different expression conditions, such as the focus of fusing different vectors in this work. Thus, Kataeva *et al*. reported that the sensitivity in previous works on predicting the solubility of recombinant proteins was much lower than the specificity [25].

**Figure 1:**
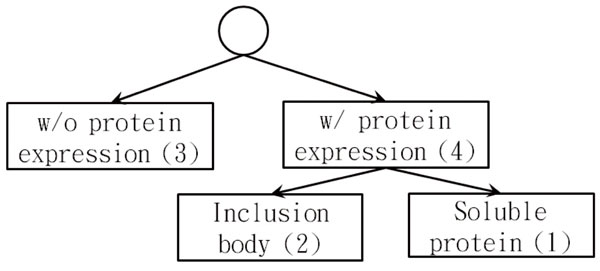
**Hierarchical structure comprising three expression levels in SDS-PAGE**. The hierarchical structure consists of three expression levels; i.e., soluble fraction, inclusion fraction, and non-expression, in SDS-PAGE.

To the best of our knowledge, this is the first attempt to consider the entire cloning and expression regions as a whole than focusing only on the sequences of the desired protein as in previous works. The entire cloning and expression regions usually consist of affinity tags and desired proteins for over-expression in recombinant protein production systems. Therefore, the objective of this study is to predict the efficacy of vectors in *E. coli *for a given protein. We propose three SVM-based methods to tackle a three-class classification problem according to the expression levels in SDS-PAGE experiments. These three classes consist of soluble fraction, inclusion fraction, and non-expression, as show in Figure 1. We finally compare our three SVM-based methods to previous studies and report two case studies of applying our methods to enhance the solubility for a given protein to be over-expressed in *E. coli*.

## Methods

### Data preparation and formulation

**Figure 2:**
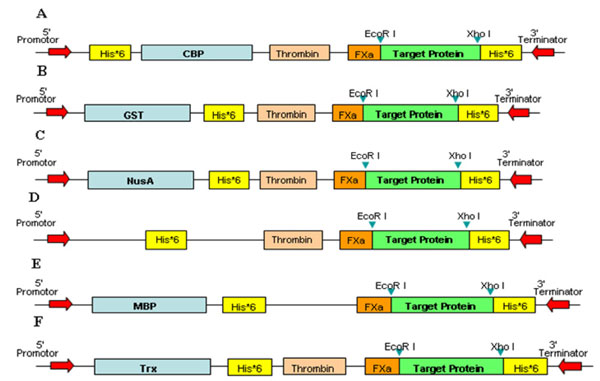
**Six fusion vectors used in HTP systems**. Cloning and expression regions of the six expression vectors and corresponding insertion locations of target proteins were used in this work. Recombinant fusion vectors were named by six fusion tags; i.e., (A) calmodulin-binding peptide (CBP), (B) glutathione S-transferase (GST), (C) N utilization substance A (NusA), (D) Histidine (His), (E) maltose-binding protein (MBP), and (F) thioredoxin (Trx).

High-throughput (HTP) protein productions were conducted to screen the over-expressions of target proteins. The system of HTP and parallel approaches in protein expression included six different fusion protein expression vectors and two universal restriction sites in *E. coli*. The expression results consisted of three levels after the treatment of denaturing SDS-PAGE. One hundred and twenty one target genes were recruited from our core facility of recombinant protein production [26]. The target genes cover a wide variety of species from virus, bacteria, mouse to human. The lengths of target genes varied from 144 to 3162. That is, the lengths of final translation proteins after being cleaved from recombinant fusion proteins varied from 48 to 1054. Given a target gene, DNA products were generated with 5' EcoRI and 3' XhoI sticky ends, and then cloned into six expression vectors in parallel. The structures of six different fusion constructs were shown in Figure 2. Fusion vectors were named by their corresponding fusion tags; i.e., CBP, GST, NusA, His, MBP, and Trx. To over-express highly soluble recombinant proteins, six different fusion constructs for each target gene were transformed into *E. coli *under the same standard experimental conditions, as well as in parallel. Host strains of *E. coli *used in this study were JM109(DE3) and BL21-CodonPlus(DE3). Host cells were harvested and lysed in 96-well plates. After centrifugation, SDS-PAGE experiments were used to separate proteins to determine expression levels. In addition, Western blot was used to further verify expression results in SDS-PAGE experiment. Finally, in a parallel analysis of protein solubility, each target gene was identified as soluble fraction, inclusion fraction, or non-expression with respect to different fusion protein vectors. In this way, 726 scenarios, 121 target genes with six different fusion vectors, were obtained. There were 231, 236, and 259 cases for soluble fraction, inclusion fraction, and non-expression, respectively (cf. Table 1).

**Table 1:** Data distribution of three expression levels in six fusion vectors. 726 cases comprised 121 genes fused into six fusion vectors generated from HTP systems.

Labels\Vectors	CBP	GST	His	MBP	NusA	Trx	Total
Soluble fraction	14	34	21	59	66	37	231
Inclusion fraction	52	37	48	28	24	47	236
Non-expression	55	50	52	34	31	37	259

Total	121	121	121	121	121	121	726

### Recombinant fusion protein solubility modelling

As shown in Figure 1, three expression levels of recombinant proteins in SDS-PAGE experiments were soluble fraction, inclusion fraction, and non-expression. Hence, after screening solubility of 121 target proteins in six different fusion vectors, the model of expression and solubility of entire recombinant proteins, including given genes and fusion vectors, was formulated as a three-class classification problem. In this work, three SVM-based methods were proposed to tackle a three-class classification problem in three different aspects of using the hierarchical structure. Furthermore, based on our experimental data of over-expression of given genes in different fusion vectors in *E. coli*, we considered entire sequences of recombinant expressed proteins instead of only sequences of target protein in previous works. Altogether, these three SVM-based methods, i.e. ***flatSVM ***, ***nestSVM ***, and ***hierSVM ***, predict each scenario, including a given gene and corresponding fusion vector, as one of the expression levels in SDS-PAGE experiments. Because *F*_1 _measure is a frequently used parameter in a multi-class classification problem, it was employed to compare the performance among three proposed SVM-based methods. Precision-Recall Curve (PRC) and Receiver Operating Characteristic (ROC) were used to compare performance of our methods to previous works. The following sections describe feature extraction and our three proposed methods in more detail.

### Feature generation

Before training SVMs, feature extraction was applied to generate fixed length feature vectors from entire recombinant expression regions. Two major steps in recombinant protein productions in *E. coli*; i.e., transcribing messenger RNAs (mRNAs) of cloning and expression regions, and translating proteins of recombinant fusion vectors, were considered. The major factors were correlated to mRNA expression and stability, codon usage in *E. coli*, solubility of whole fusion vectors, and Post-Translational Modifications (PTMs) on recombinant proteins. Hence, based on nucleotide and protein levels, entire cloning and expression regions were used to retrieve potential features for predicting transcription efficacy and solubility propensity of recombinant fusion proteins in *E. coli*. For mRNA expression and stability, 84 k-mer features where k = 1, 2, and 3, along with transcribed mRNA length for each recombinant fusion gene.

Guanine-cytosine content (GC-content) calculated from nucleic acid sequences of recombinant fusion genes were also taken into account. For protein expression, codon usage bias in *E. coli *could be a key factor of determining the efficiency of translation. Moreover, based on the reconstruction of phylogenetic tree, Elena *et al*. have concluded that B and K12 strains are the most closely related ones in *E. coli *[27]. Consequently, in this study, Codon Adaptation Index (CAI) has been calculated based on codon preference in *E. coli *K12 strain by EMBOSS package [28]. As described in previous works, 6 and 444 sequence-independent and non-redundant features used in [13] and [22] were extracted, respectively. Features used in most previous works were extracted only from sequences of target proteins to predict solubility. However, different results having been observed by using different fusion vectors were considered in this study. Instead of considering only target proteins, features in protein levels were derived from entire recombinant fusion proteins. It is known that PTMs rarely occur in *E. coli*. However, for higher eukaryotic proteins, some critical steps of correct protein folding may be related to certain PTMs. Therefore, in the present work, predicted PTMs on entire recombinant fusion proteins were further considered. 71 PTMs predicted by AutoMotif [29] were used to reveal critical steps of PTMs on recombinant fusion proteins to aid prediction of recombinant fusion protein solubility. In addition, compared to solubility-related features used in the previous works, isoelectric point and other 8 peptide statistics calculated by EMBOSS.iep and EMBOSS.pepstats were included [28]. Finally, 617 features for each instance vector were generated (cf. Table 2).

**Table 2:** Feature index used in this study.

Feature Type	Description	#(Feature)
Nucleotide	≤3-mer	84
Nucleotide	nt Seq Length	1
Nucleotide	GC Content	1
Code Preference	Codon Adaptation Index	1
Amino Acid	Wilkinson and Harrison (1991)	6
Amino Acid	Idicula-Thomas *et al*. (2006)	444
Amino Acid	isoelectric point	1
Amino Acid	peptide statistics	8
PTMs	Plewczynski *et al*. (2005)	71

	Total	617

617 features were extracted from an entire recombinant fusion protein. They were divided into two groups with respect to nucleotide or protein levels. The first 87 features were generated from nucleic acid sequences of entire recombinant fusion genes. The rest 530 features were retrieved from protein sequences.

### Three SVM-based methods for classification

Support Vector Machines (SVMs) are one type of machine learning techniques used for classification and regression originally developed by Vapnik based on the statistical learning theory [19]. SVMs search for a hypothetically unique and optimal hyperplane to distinguish data by maximizing the margin. By cooperating with kernel functions, SVMs map original data that are non-linearly separable in input space into a high-dimensional feature space. In this paper, expression level prediction of recombinant fusion proteins was formulated as a three-class classification problem; i.e., soluble, insoluble, and non-expression. After scaling features generated by feature generation, all features in instance vectors were normalized to zero mean and Standard Deviation (SD) to 1. Here, three SVM-based methods were proposed to deal with the three-class classification problem. With respect to different aspects of considering the hierarchical structure formed by expression levels of recombinant fusion proteins, instance vectors were treated as flat, nested, or hierarchical ones. Three SVM-based methods were named as ***flatSVM ***, ***nestSVM ***, and ***hierSVM ***, respectively. LIBSVM [30] were used to implement all core algorithms in this research. According to the characteristics of features, the radial basis function (RBF) kernel implemented in LIBSVM was used because of its advantages on dealing with the most cases of numerical data. In the present work, all instance vectors were stratified sampling among three classes. In each class, the same proportion is present in each random partition to divide instances into m parts. In training and validation, k-fold Cross-Validations (CVs) were applied to the m-1 parts of instance vectors. The last part was used as test. The procedure of training and testing was repeated for n times. Finally, performance results of these n repeats were averaged and their corresponding SDs were measured.

#### flatSVM

According to the hierarchical structure as shown in Figure 1, we treated this binary-tree taxonomy as a flat one. Generally, one-against-one (1vs1) and one-against-the rest (1vsAll) are two commonly used strategies on dealing with multi-class classification problems. However, as reported by Hsu *et al*., 1vsAll strategy may get a comparable performance as 1vs1 strategy, but it takes much more time on training [31]. Therefore, considering the cost of training time, we decided to use 1vs1 strategy instead of 1vsAll strategy in this work. As mentioned in Feature Generation, instance vectors were derived with 617 features from entire recombinant fusion regions and 726 instance vectors were stratified by labels and divided into ten parts randomly. Along with their corresponding labels of expression levels, 652 instance vectors were used on training and validation by 10-fold CVs. By using three 1vs1 classifiers, the prediction class of an instance vector was determined by a majority voting. The other unseen 74 instance vectors in training and validation were applied to evaluate the performance of trained classifiers. By repeating the same procedure of training and testing in ten times, an average and SD were calculated for these ten CV results. All programs were implemented and associated with LIBSVM package [30].

#### nestSVM

Following the procedure of transcribing and translating a recombinant fusion protein in *E. coli*, the hierarchical structure was divided into two steps. The first step was related to protein expression and the second was associated with the solubility of expressed proteins. First, mRNA expression and stability for a recombinant fusion gene and codon preference in *E. coli *were the major factors. For second step, solubility related features to test whether an expressed protein could be folded correctly as a soluble one in *E. coli *were applied. Based on the divide-and-conquer conception, a stepwise method, ***nestSVM ***, was proposed to undertake the three-class classification problem by training corresponding classifier for each step. This way, two binary classifiers were trained with distinct sets of features to predict whether a recombinant fusion gene could be expressed and whether an expressed recombinant fusion protein would be soluble in *E. coli*. For protein expression, a binary classifier was trained to distinguish whether a recombinant fusion gene could be expressed as a protein in *E. coli *by focusing on features derived from nucleic acid sequences. 84 k-mer frequency features, length, GC-content, and CAI were derived from entire recombinant fusion genes used in first binary classifier. Similar to ***flatSVM ***, 652 instance vectors with 87 features were applied to 10-fold CVs on training and validation of the first classifier. However, for the first classifier, instance vectors labelled with soluble and insoluble were treated as one class. Furthermore, for predicting solubility of expressed recombinant fusion proteins in *E. coli*, the second classifier was trained by the other non-overlapping 530 features in protein level. For a perfect case, all instance vectors of soluble and insoluble used in first step were promoted to train the second classifier. Hence, 207 soluble cases and 212 insoluble cases were used for 10-fold CVs for training and validation. The second binary classifier was mainly used to determine instances between soluble and insoluble proteins. For testing the performance of ***nestSVM ***, 74 unseen instance vectors were used to predict protein expression by the first binary classifier. In the second binary classifier, it was applied to instances that were labelled as expression in the first step to predict their protein solubility. The three-class classification problem was tackled by combining two binary classifiers for predicting expression and solubility of recombinant fusion proteins in *E. coli *respectively.

#### hierSVM

For our third method, class labels were considered as attribute vectors instead of arbitrary numbers and involved the concept of hierarchical classification method [32]. Because of resource availability constraints, we did not implement the entire algorithm. Instead, we reduced it into a binary SVM classification to fit the public domain tool. The algorithm was described as follows. According to Figure 1, attribute vectors of labels were encoded as <1, 0, 0, 1>, <0, 1, 0, 1>, and <0, 0, 1, 0> to illustrate soluble, insoluble, and non-expression, respectively. Here, the first three digits in attribute vectors were associated to the original labels in the order of soluble, insoluble, and non-expression. The last digit in attribute vectors represented the common parent node of labels between soluble and insoluble proteins in class taxonomy. In other words, for the instance labelled as non-expression, the last digit will be zero for the attribute vector.

In order to reduce a hierarchical SVM classification into a binary classification, subtractions between pairs of attribute vectors were taken to implement the idea. For example, when considering an instance vector with its label as soluble, two new attribute vectors of positive cases were produced by subtracting attribute vectors of insoluble and non-expression from attribute vector of soluble, respectively. Hence, two new attribute vectors of positive cases were <1, -1, 0, 0> and <1, 0, -1, 1>. In other words, subtracting attribute vector of soluble from attribute vectors of insoluble and non-expression, generated two new attribute vectors for negative cases, therefore, two new attribute vectors of negative cases were <-1, 1, 0, 0> and <-1, 0, 1, -1>. By using tensor product ⊗ to cooperate attribute vectors of labels into instance vectors, 617 features in an instance vector were expanded to four times, i.e., 2468 features. Consider an instance vector **X**, after expanding the four new attribute vectors were generated. <**X, -X, 0, 0**> and <**X, 0, -X, X**> were positive cases and <**-X, X, 0, 0**> and <**-X, 0, X, -X**> were negative cases. Finally, for training and validation, two positive cases and two negative cases were used. For testing, 6 pairs of subtractions between attribute vectors of labels were applied to predict and averaged to decide the final prediction label. Similar to our other two methods, after a stratified selection, 652 instance vectors were used in training and validation for 10-fold CVs, and then the remaining 74 instances were applied as test cases.

### Evaluation measurement

Given a multi-class classifier, *F*_1 _measure is the proper parameter of its performance. *F*_1 _measure is calculated as(1)

where *J *is the total number of classes, *A*_*j *_is the number of instances correctly predicted as class j, *B*_*j *_is the number of instances incorrectly assigned to class *j*, and *C*_*j *_is the number of class *j *instances assigned to other classes. Taking a general case of *F*_1 _measure as example, *F *score is simply derived and represented as(2)

where *TP*, *FP*, and *FN *represent true positive, false positive, and false negative, respectively. Alternatively, the well-known representation of *F *score for a binary classification is associated with precision and recall, which are denoted as *p *and *r*, respectively. Generally, a system with good performance will assign the correct class and only the correct class, by maximizing not only precision but also recall, and then results in maximizing the *F *score.

In this work, each SVM-based classifier was used as a three-class classifier to predict expression levels of recombinant fusion proteins in *E. coli*. Hence, the performance of three proposed SVM-based classifiers were compared to each other by *F*_1 _measure. However, to compare with previous works, three proposed SVM-based classifiers were reduced to distinguish soluble from non-soluble cases, including insoluble and non-expression cases. By using *F *score, performance of our methods was compared with previous works under the same criterion. Moreover, the areas under Receiver Operating Characteristic (ROC) and Precision Recall Curves (PRC) were used to assess the performance of our methods with previous works. ROC consists of False Positive Rate (FPR) and True Positive Rate (TPR) as x and y-axes, respectively. On the other hand, PRC consists of True Positive Rate (TPR) and Positive Predictive Value (PPV) as x and y-axes, respectively. The detail calculations were as follows.(3)(4)(5)

To investigate difference between pairs of our three methods, *Student's t-test *and *Yule's Q-statistic *[33] were conducted to demonstrate the relationship of diversity. The *Yule's Q-statistic *is defined as(6)

where *N*^*ij *^is the number of instances in the test instances, classified correctly (*i *= 1) or incorrectly (*i *= 0) by the first classifier, and correctly (*j *= 1) or incorrectly (*j *= 0) by the second classifier. After calculation, the range of *Q *varies from -1 to 1. For statistically independent classifiers, the calculation will be equal to zero. On one hand, the positive value of *Q *indicates that classifiers tend to identify the same instances correctly. On the other hand, classifiers commit errors on different instances will have a negative *Q*.

## Results and discussion

### Predictive performance of our proposed SVM-based methods

In order to investigate expression levels of target proteins cloned into individual fusion vectors, six three-class classifiers were trained and assessed by applying ***flatSVM ***. Six hundred and seventeen features for each target gene associated with individual vector were generated as mentioned in Feature Generation. The process of training and testing were used to 121 target genes for each vector to train a three-class classifier. All target genes were divided into five parts. Four out of five parts were used to train a classifier by 5-fold CVs. The last part of 25 target genes was tested by the classifier trained in previous step. As elucidated in Table 3, the best performance occurred in the classifiers based on GST vector for the average *F*_1 _measure. The result showed that the classifier of GST vector outperformed other five classifiers trained with respect to other vectors on classifying instances into three expression levels correctly. However, the performance of six individual classifiers trained independently per vector was still far from satisfactory.

**Table 3:** Performance evaluation of six individual classifiers with respect to six vectors.

Vectors\Measure	***F***_**1 **_** measure**
CBP	0.3509 ± 0.0372
GST	0.4323 ± 0.1515
His	0.4016 ± 0.0874
MBP	0.3746 ± 0.1017
NusA	0.3297 ± 0.0695
Trx	0.2854 ± 0.0609

For *F*_1 _measure, each individual classifier must correctly identify each instance into its real class

Instead of taking experimental data separately to train six classifiers with respect to each vector, all data were considered together in the following experiments. For our three proposed SVM-based methods, the predictive performance was shown in Table 4. We also showed the performance of AdaBoost as our baseline performance. Overall, ***flatSVM ***with high *F*_1 _measure, *F *score, and accuracy seems to outperform other methods. Using *Student's t-test*, we investigated the pair wise relationship of three proposed methods with respect to accuracy. We showed in Table 5 that only ***flatSVM ***and ***nestSVM ***resulted in statistical significance with a p-value less than 0.05.

**Table 4:** Performance evaluation of three proposed methods

Avg.\Methods		flatSVM	nestSVM	hierSVM	AdaBoost
*F*_1 _measure		0.7791 ± 0.0606	0.6989 ± 0.0578	0.7466 ± 0.0464	0.7241 ± 0.0287
*F *score		0.7551 ± 0.0719	0.7068 ± 0.0600	0.7000 ± 0.0498	0.7075 ± 0.0442
	Recall	0.7833 ± 0.0998	0.7875 ± 0.0747	0.7083 ± 0.0900	0.7000 ± 0.0852
	Precision	0.7397 ± 0.1027	0.6466 ± 0.0795	0.7015 ± 0.0718	0.7240 ± 0.0567
Accuracy		0.8351 ± 0.0488	0.7865 ± 0.0528	0.8041 ± 0.0320	0.8135 ± 0.0253

For *F*_1 _measure, each individual classifier must correctly identify each instance into its real class. In contrast, *F *score just focuses on measuring the rate of correctly identifying soluble fraction instances from non-soluble ones, i.e. the class of soluble fraction versus the other two classes.

**Table 5:** Student's t-test in accuracy.

T-test in Accuracy (pvalue)	*flatSVM*	*nestSVM*
***nestSVM***	0.045*	
***hierSVM***	0.1075	0.3789

*Student's t-test *in accuracy was performed to 10 repeats of evaluation procedures among three proposed methods. ***flatSVM ***method performed significantly better than ***nestSVM ***method with a p-value less than 0.05.

### Comparisons with solubility prediction methods

**Figure 3:**
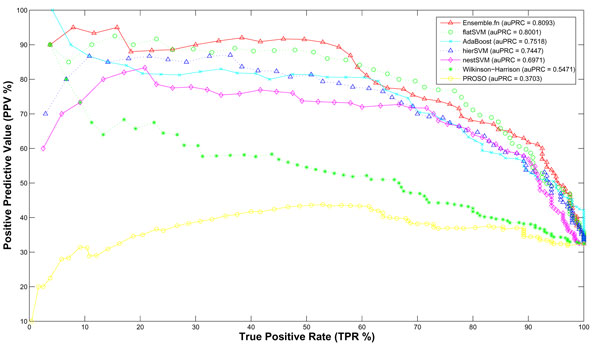
**Comparative analysis with PRC curves**. The PRC Curves from the comparative analysis of solubility prediction methods.

**Figure 4:**
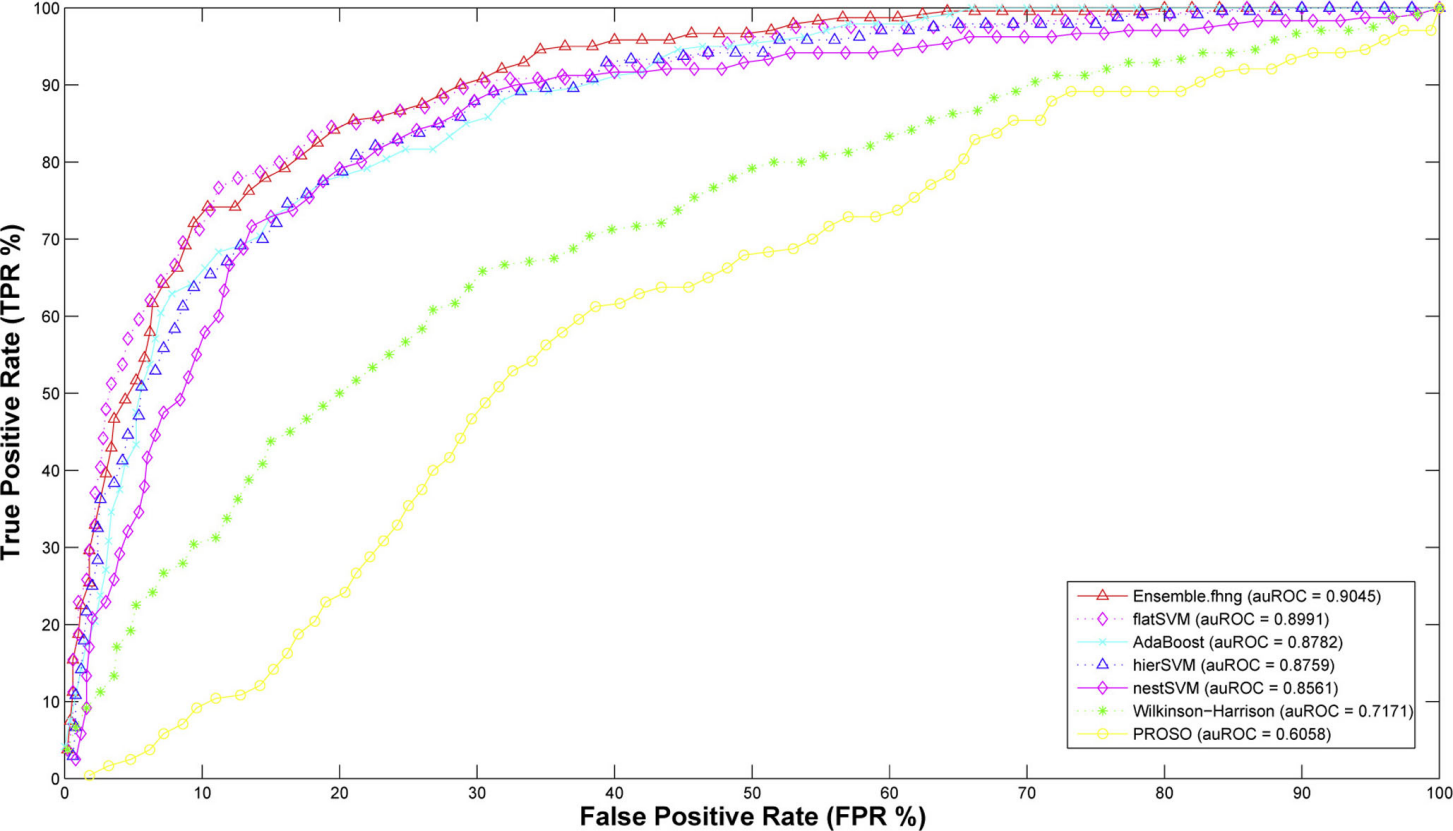
**Comparative analysis with ROC curves**. The ROC Curves of the comparative analysis of solubility prediction methods.

To compare our proposed methods with other previously published predictors, our methods were reduced to a binary classifier of predicting solubility of proteins from non-soluble ones, which included insoluble and non-expression. As we have discussed, the widely used and available predictors are Wilkinson-Harrison [13] and PROSO [23]. By submitting each recombinant fusion proteins to their provided web servers, calculated results were acquired. As illustrated in Figure 3 for PRC, each point in the plot was an average of 10 repeats of test cases for all methods. To compare two methods in previous works, Wilkinson-Harrison method achieved a better result than PROSO by 0.5471. According to the claim of PROSO, correct evaluation occurred only when target proteins were without trans-membrane segment. Hence, after checking proteins in our dataset by TMHMM [34], the bad performance of PROSO would be because of the existence of trans-membrane segments in recombinant fusion proteins that were not removed. As shown in Figure 4, the area under ROC curve of PROSO is 0.6058, which is closer to 0.781 reported in the paper. Compared our three methods to others, the best result of our proposed method is ***flatSVM ***, which outperformed by achieving the auPRC and auROC of 0.8001 and 0.8891, respectively.

We used the feature selection package provided in LIBSVM to measure the importance of the features. We found that if we remove those less important features from our feature set, it will result in a lower accuracy. For instance, after doing feature selection and keeping 37 the most important features, the testing accuracy will dramatically decrease from 87.84% to 45.95% in one of the best cross-validation model in ***flatSVM ***. Hence, we decided to keep all 617 features to maintain the performance.

### Model ensemble of proposed methods

To investigate the diversity of our proposed methods, we calculated *Yule's Q-statistic *between pairs of proposed methods. The results in Table 6 indicate that each method permit to train a classifier in a partial un-correlation. This outcome could motivate the importance to combine different methods into an ensemble one. As shown in Figure 3 for PRC, ensemble models between ***flatSVM ***and ***nestSVM ***resulted in a higher auPRC of 0.8093. As shown in Figure 4, ensemble models among all methods achieved 0.9045 in auROC.

**Table 6:** Yule's Q-statistic between proposed methods.

Q-statistic	*flatSVM*	*nestSVM*	*hierSVM*
***nestSVM***	0.9022		
***hierSVM***	0.9424	0.8569	
***AdaBoost***	0.8527	0.7740	0.7979

*Yule's Q-statistic *was performed to proposed methods. It shows that ***flatSVM ***and ***hierSVM ***tend to make similar mistakes and correct classifications.

### Computational simulation of enhancing solubility by limit mutations or optimal codons

In biotechnology perspective, to improve the solubility of target proteins, some laboratory workers may mutate few nucleotides in target genes without affecting their corresponding translational proteins. Moreover, according to the codon preference in *E. coli*, they synthesize whole nucleotide sequences based on the amino acid sequences. Here, we designed two computational simulations to enhance the solubility of recombinant proteins via our prediction classifier. First, while considering a proper number of mutation sites in biological laboratories, we limited the maximum steps of mutation sites to five. Meanwhile, to effectively reduce the search space, we employed a beam search for narrowing down the search to the top five potential candidates to be soluble forms, which were predicted by our classifier. On the other hand, we designed the second simulation by using preferential codons in *E. coli *to synthesize entire nucleotide sequences of target proteins. By inputting these synthesized nucleotide sequences into our classifiers, we want to investigate whether any changes in expression levels will occur.

**Figure 5:**
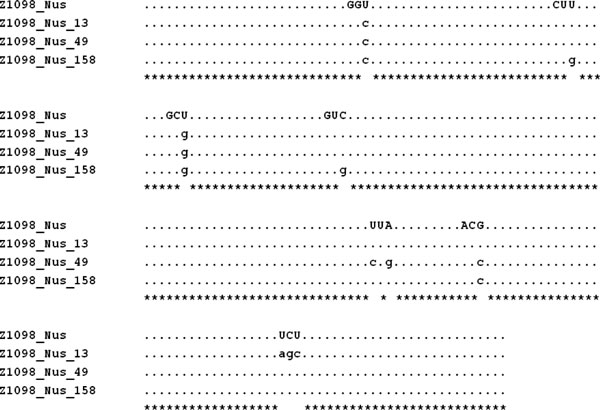
**Multiple sequence alignment for one mutation case**. The result of Multiple Sequence Alignment (MSA) for one case which was predicted as a soluble form after mutating 5 nucleotides was presented.

In our data set, there are totally 259 scenarios, including a given gene and its corresponding vector, resulted in a non-expression level. By mutating few nucleotides or by synthesizing nucleotide sequences based on the codon preference in *E. coli*, we tried to recover these 259 cases from non-expression to soluble fraction. For the first part, there are two cases predicted by our classifier to change their expression level to soluble forms after one and five steps, respectively. As shown in Figure 5, this case was originally in the non-expression level, but was predicted as a soluble from after mutating five bases. For the simulation of using synthesized nucleotide sequences, there were totally 31 out of 259 cases that were successfully predicted as soluble fractions as well as nine out of 259 cases were predicted as insoluble fractions.

## Conclusion

Since the days when Wilkinson and Harrison started applying statistical and computational approaches on studying the solubility prediction for a given protein over-expressed in *E. coli *[13], a large number of researchers developed variety of methods on extracting the important factors to affect the solubility of recombinant proteins. In this study, we developed three SVM-based methods of predicting three expression levels based on SDS-PAGE experiments for a target protein in a corresponding vector in *E. coli*. Unlike most previous works of omitting the cases of no protein expressions in *E. coli*, this work is the first attempt to tackle a three-class classification problem of distinguishing the expression level for a desired protein in SDS-PAGE experiments. Moreover, according to the observation from our experimental data, a given protein could result in different expression levels when being over-expressed in different vectors in *E. coli*. Therefore, this work is the instance of encompassing the entire cloning and expression regions. By using our classifiers, the prediction results could help biologists effectively and efficiently choose among different vectors to gain soluble recombinant proteins in *E. coli*. Additionally, in biotechnology perspective, by mutating few nucleotides or by synthesizing optimal sequences according to the codon preference in *E. coli*, our prediction methods also provide effective ways to enhance the solubility of target proteins.

## Competing interests

The authors declare that they have no competing interests.

## Authors' contributions

All authors conceived the project and design. YS and PL prepared the data. WC implemented the algorithms, performed the computational experiments, and analyzed the results. UY partly initiated the design of computational analyses. WC, WL, and CH wrote the paper. All authors read and approved the document.
